# Multimodal Sensor Fusion for Non-Destructive Tea Quality Evaluation: Deep Learning-Enabled Methods, Applications, and Challenges

**DOI:** 10.3390/foods15101810

**Published:** 2026-05-20

**Authors:** Xinyu Hu, Meng Zhang, Biyue Yang, Yuefei Tao, Wei Wei

**Affiliations:** School of Agricultural Engineering, Jiangsu University, Zhenjiang 212013, China

**Keywords:** tea quality, non-destructive evaluation, multimodal sensing, deep learning, data fusion, sensor complementarity

## Abstract

Tea quality evaluation is increasingly moving from subjective sensory assessment and destructive laboratory analysis toward rapid, non-destructive, and data-driven approaches. This review summarizes recent advances in multimodal sensing integrated with deep learning for tea quality evaluation, with emphasis on sensor complementarity, data-fusion strategies, representative applications, and deployment-related limitations. Major sensing modalities, including machine vision, near- and mid-infrared spectroscopy, Raman and fluorescence spectroscopy, hyperspectral imaging, and electronic nose/electronic tongue systems, are discussed in relation to their ability to characterize appearance, chemical composition, aroma, flavor, processing status, and safety-related attributes. Applications are examined for quality grading, chemical composition prediction, aroma and flavor characterization, fermentation monitoring, and safety-related extensions across representative tea products, including green tea, black tea, dark tea, matcha, and jasmine tea. Overall, multimodal approaches can outperform single-sensor systems only when the selected modalities provide complementary, rather than redundant, information layers. However, practical translation remains constrained by small and weakly standardized datasets, insufficient external validation, sensor instability, limited model transferability, high computational cost, and insufficient interpretability. Future research should prioritize standardized datasets, leakage-free validation protocols, interpretable multimodal modeling, truly independent external validation, interoperable multi-sensor platforms, and lightweight deployable models.

## 1. Introduction

Tea is one of the most widely consumed beverages in the world, and its quality and safety directly influence consumer acceptance and public health. Traditionally, tea evaluation has relied on sensory inspection by trained assessors and on laboratory-based physicochemical analysis. Sensory assessment considers appearance, aroma, and taste, but its reproducibility is often limited by assessor experience and subjectivity [1,2]. Instrumental methods such as gas chromatography–mass spectrometry (GC–MS) provide accurate chemical information, yet they are often time-consuming, costly, and destructive. For example, aroma profiling in black tea typically depends on GC–MS-based identification and quantification, whereas the determination of bioactive compounds in green tea often requires labor-intensive extraction and wet-chemistry procedures [3,4]. These limitations make conventional approaches increasingly inadequate for a modern tea industry that requires rapid, objective, and non-destructive quality control.

With recent advances in sensor technology and artificial intelligence, multimodal sensing has emerged as a promising route for tea quality assessment [5,6,7]. In this context, multimodal sensing refers to the acquisition of complementary information from multiple sensor types, followed by data integration to characterize tea quality more comprehensively [8,9]. Common modalities include machine vision, which captures appearance-related traits such as shape, size, and color uniformity—for example, by converting RGB images into HSV or Lab space to quantify browning during fermentation [10]; spectroscopic methods, including near-infrared (NIR), mid-infrared, Raman, and fluorescence spectroscopy, which probe chemical composition and molecular structure and enable rapid prediction of constituents such as polyphenols and amino acids [11,12,13]; and electronic nose/electronic tongue systems, which emulate human olfactory and gustatory perception through sensor arrays for aroma, flavor, origin, and grade discrimination [12,14,15]. When these complementary data streams are coupled with modern pattern-recognition algorithms, they enable more objective and robust tea evaluation.

Deep learning has become a central tool in intelligent food-quality analysis [16,17,18]. By learning hierarchical nonlinear representations from raw or minimally processed data, deep models can outperform conventional empirical indices and shallow machine-learning approaches. Convolutional neural networks (CNNs) are particularly effective for image-based tea grading [19], whereas one-dimensional CNNs and recurrent neural networks (RNNs) are well suited to spectral or temporal sensor signals, such as fermentation dynamics and flavor evolution. Attention mechanisms and residual architectures can further improve feature selection and model stability under limited-sample conditions [20]. Nevertheless, model performance still depends heavily on dataset quality and representativeness, underscoring the need for larger, publicly accessible datasets covering multiple tea types, origins, and processing conditions.

Several reviews have addressed tea quality evaluation from the perspectives of analytical techniques, sensory methods, or specific sensing tools. More recently, Wu et al. [21] systematically reviewed deep-learning applications in tea quality monitoring across cultivation, processing, and product evaluation. In contrast, the present review focuses specifically on multimodal, non-destructive tea quality evaluation, with particular emphasis on sensor complementarity, fusion strategies, representative tea products, and deployment-related challenges. We reorganize the literature according to a quality-attribute-driven framework that links target quality attributes, target substance groups, sensing modalities, fusion strategies, deep-learning architectures, and deployment constraints. We aim to clarify why multimodal sensing can outperform single-sensor systems and what conditions are required for its industrial translation.

## 2. Framework of Multimodal Sensing and Deep Learning for Tea Quality Evaluation

### 2.1. Tea Quality Attributes and Target Substance Groups

Tea quality is inherently a multidimensional concept and cannot be fully characterized by a single sensory attribute or a single physicochemical parameter. For finished tea products, appearance, color, liquor color, aroma, taste, mouthfeel, processing status, and key chemical composition jointly determine overall quality performance. The major target substance groups directly associated with these quality attributes include tea polyphenols and catechins, caffeine, free amino acids, particularly theanine, soluble sugars, pigments, volatile organic compounds (VOCs), and moisture-related indicators. For fermented or post-fermented teas, such as black tea and dark tea, dynamic changes in theaflavins, thearubigins and their oxidative polymerization products, fermentation-derived volatiles, and taste-active compounds are also important bases for evaluating fermentation degree and final product quality [12,13,14].

From the perspective of multimodal sensing, tea quality attributes can be mapped onto several interrelated but non-equivalent information dimensions. Appearance, leaf shape, color, and uniformity are mainly reflected in visual and textural information; changes in polyphenols, caffeine, amino acids, moisture, and pigments are more directly associated with molecular responses involving absorption, scattering, fluorescence, or Raman signals; aroma quality mainly depends on VOC fingerprints; taste is closely related to soluble taste-active compounds in tea infusion and their electrochemical response patterns. Processing states, such as fixation, rolling, fermentation, drying, scenting, and aging, are manifested as the synchronous evolution of visual, spectral, gaseous, and environmental parameters [19,20]. Therefore, the premise of multimodal tea evaluation is not merely the simultaneous use of multiple sensors, but rather the prior clarification of the correspondence among target quality attributes, target substance groups, measurable information dimensions, and sensing modalities. In the strict sense, non-destructive detection should refer to analytical methods that complete measurement without compromising the original integrity of the sample. However, some spectroscopic, electrochemical, or enhanced sensing methods in tea research still require grinding, homogenization, infusion preparation, surface-enhanced substrates, or other pretreatment steps. Therefore, this review distinguishes strictly non-destructive methods from minimally destructive or sample-preparation-assisted rapid sensing approaches when necessary (Table 1).

**Table 1:** Mapping of Tea Quality Attributes, Target Indicators, Sensing Modalities, and Fusion Strategies.

Quality Attribute	Target Indicators	Dominant Modalities	Complementary Modalities	Suitable Fusion Strategies	Main Limitations
Appearance and color	Leaf shape/strip appearance, particle size, color uniformity, liquor color, surface defects	Machine vision, microscopic imaging	HSI/MSI, GLCM texture features	Early/intermediate fusion	Sensitive to illumination, background, and sample stacking
Intrinsic chemical composition	Tea polyphenols, catechins, caffeine, free amino acids, soluble sugars, moisture	NIR/MIR	Raman/SERS, HSI, fluorescence	Intermediate fusion	Difficult cross-instrument transfer and pronounced matrix effects
Aroma quality	VOC fingerprints, floral/fresh/roasted aroma, scenting intensity	Electronic nose, colorimetric sensor array, GC–IMS	GC–MS, Vis-NIR/HSI	Late/intermediate fusion	Sensor drift and insufficient specificity for low-abundance key aroma compounds
Taste and mouthfeel	Freshness/umami, bitterness, astringency, mellow mouthfeel, soluble taste-active compounds	Electronic tongue, electrochemical sensor array	NIR, FT-NIR, reference chemical analysis	Intermediate/late fusion	Electrode fouling and strong matrix effects in tea infusion
Processing status	Fixation/drying endpoint, fermentation degree, aging stage	HSI, NIR, electronic nose	IoT-based environmental sensing, machine vision	Temporal intermediate/late fusion	Difficulty in continuous annotation and high requirements for temporal synchronization
Safety-related extensions	Screening of pesticide residues, contaminants, adulteration, or abnormal samples	SERS, fluorescence, HSI	NIR, imaging, mass spectrometry confirmation	Late fusion	Diverse targets, insufficient databases, and some methods are not strictly non-destructive

HSI, hyperspectral imaging; MSI, multispectral imaging; GLCM, gray-level co-occurrence matrix; NIR, near-infrared spectroscopy; MIR, mid-infrared spectroscopy; SERS, surface-enhanced Raman spectroscopy; VOCs, volatile organic compounds; GC–IMS, gas chromatography–ion mobility spectrometry; GC–MS, gas chromatography–mass spectrometry; Vis-NIR, visible–near-infrared spectroscopy; FT-NIR, Fourier-transform near-infrared spectroscopy; IoT, Internet of Things. Fusion strategies listed in the table indicate recommended or commonly applicable approaches and are not mutually exclusive.

### 2.2. Complementarity Among Sensing Modalities

Multimodal sensing captures different information dimensions of the same sample through multiple sensors, thereby providing a more comprehensive representation than a single modality [20]. Compared with single-modal approaches, the advantage of multimodal methods lies not merely in “collecting more data”, but in the fact that different sensors correspond to different layers of quality-related information. As a result, they can form complementary relationships in complex samples, thereby improving quality characterization, model robustness, and cross-scenario adaptability [8,9,20].

Machine-vision-based modalities mainly characterize external attributes, including leaf shape, strip morphology, particle size, color uniformity, liquor color, and surface defects [19,22]. Their strength lies in rapid, non-contact acquisition and strong interpretability for grading tasks. When image features are extended from simple RGB color descriptors to HSV or Lab representations, or further to statistical texture descriptors, visual sensing becomes more sensitive to structural heterogeneity and subtle process-induced changes. In this context, GLCM-based texture descriptors can serve as interpretable complements to deep visual features, especially for strip tea, granular tea, and matcha powder, where surface morphology contributes materially to quality evaluation [23,24].

Spectroscopic methods mainly characterize internal chemical composition and molecular structure. NIR and MIR are most commonly used in rapid quantitative tea analysis to predict moisture, tea polyphenols, caffeine, free amino acids, and other major constituents [11,12,13,25,26,27,28]. Raman spectroscopy and SERS provide more molecule-specific vibrational fingerprints and are suitable for the rapid identification of characteristic molecules or trace targets [27]. Fluorescence spectroscopy and fluorescence hyperspectral imaging can capture differences in excitation–emission responses caused by certain components or surface residues [26]. HSI integrates spatial and spectral information, enabling simultaneous characterization of “component content” and “spatial distribution”; therefore, it is particularly advantageous for visualizing chlorophyll, moisture, pigment distribution, powder uniformity, and local defects [20,25,29].

Olfactory and gustatory sensor systems represent another critical layer of complementarity. In this review, olfactory sensors mainly refer to MOS-based electronic noses, colorimetric sensor arrays, and GC–IMS-assisted volatile-fingerprint systems that capture VOC-related aroma profiles [2,14,30]. Taste sensors mainly refer to electronic tongues and electrochemical sensor arrays that reflect the electrochemical response patterns of amino acids, catechins, caffeine, soluble taste-active compounds, and related matrix effects in tea infusion [12,14,15]. These modalities are especially useful when chemical composition alone cannot adequately explain perceived aroma intensity, freshness, bitterness, astringency, or processing degree.

**Figure 1:**
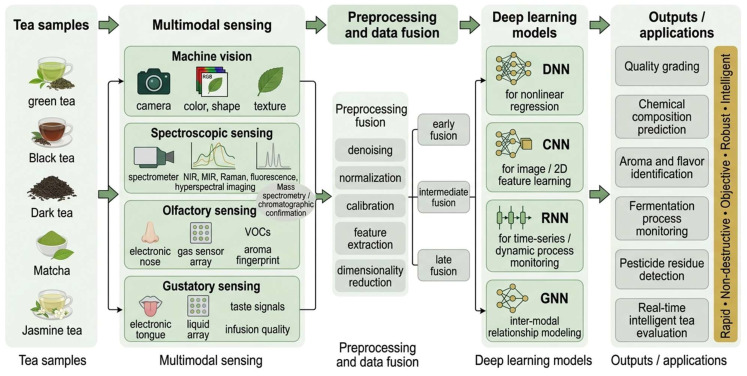
Integrated application schema of multimodal sensing and deep learning for tea quality evaluation. Abbreviations: DNN, Deep Neural Networks; CNN, Convolutional Neural Networks; RNN, Recurrent Neural Networks; GNN, Graph Neural Networks.

Therefore, the true value of multimodal systems lies in complementarity rather than redundant accumulation. Visual modalities are effective for characterizing appearance and color, but they cannot directly quantify theanine or caffeine; NIR is suitable for rapid prediction of overall chemical composition, but it is insufficiently sensitive to subtle aroma differences; Electronic noses can capture volatile profiles, but they cannot directly explain taste; Electronic tongues can reflect taste-related electrochemical patterns, but they do not provide information on leaf strip morphology or aroma purity. Precisely because these blind spots are offset across modalities, the integration of image, spectral, olfactory, and gustatory information can better approximate the comprehensive decision-making logic used in sensory evaluation and quality control (Figure 1). For example, image–spectral information fusion has been used to improve the prediction of chlorophyll distribution during tencha/matcha drying [20,29], whereas the fusion of olfactory and gustatory sensing signals can enhance the accuracy of oolong tea variety discrimination [31]. These findings indicate that the decisive condition under which multimodal approaches outperform single-sensor systems is whether the complementary modalities correspond to different but related layers of quality information.

### 2.3. Data Fusion Strategies for Heterogeneous Tea-Sensing Data

Early fusion refers to the direct concatenation of raw signals or manually extracted features before modeling. For example, color parameters, texture vectors, spectral variables, electronic-nose responses, and electronic-tongue signals can be combined into a unified feature matrix and then input into PLS, SVM, random forest, or neural network models [24,31]. Its advantages are straightforward implementation and suitability for small-sample studies. However, its limitations are also evident: different modalities often differ substantially in dimensionality, scale, noise level, and sampling frequency. High-dimensional spectral variables may overwhelm low-dimensional visual or sensor-array response features, causing the model to become biased toward a particular modality. Therefore, normalization, variable selection, modality weighting, and outlier control are key preprocessing steps for early fusion.

Intermediate fusion, also known as feature-level deep fusion or representation-level fusion, is currently one of the most suitable strategies for multimodal tea evaluation. Its basic principle is to first extract features using modality-specific encoders and then couple these features within a shared latent space. For example, CNNs can be used to extract spatial features from images or HSI data, 1D-CNNs to extract local spectral features, and RNNs or temporal modules to capture dynamic features during fermentation or drying. These features can then be integrated through concatenation layers, attention modules, bilinear pooling, graph-structured associations, or transformer-based cross-modal interactions [32,33,34]. This approach preserves the structural characteristics of image, spectral, and sensor-array signals while making it easier to identify, through attention weights, which modality, wavelength, region, or time point is most critical for the final prediction.

Late fusion integrates the output-layer results after each modality has been modeled separately. Typical forms include weighted averaging, voting, stacking, Bayesian ensemble methods, or rule-driven multi-level decision-making. This strategy is particularly suitable for industrial deployment, because vision systems, NIR instruments, electronic noses, and electronic tongues in factory settings are often not sampled synchronously, and their maintenance cycles and failure probabilities also differ. Under such conditions, modular late fusion is more robust than full joint training and makes it easier to maintain system operation when one modality is missing, drifting, or temporarily unavailable. At the same time, late fusion offers advantages in interpretability, because the output contribution of each submodel can be examined independently.

For multimodal tea-sensing data, future optimization of fusion strategies should not focus only on how to improve accuracy in a single experiment, but should also emphasize the engineering usability of heterogeneous data. This includes cross-modal normalization, reliability-based modality weighting, robust training under missing-modality conditions, cross-device calibration and model transfer, external validation-set design, and the incorporation of calibration transfer and instrument standardization into multimodal pipelines, thereby reducing shifts caused by differences among instruments, batches, and production origins [24,31].

### 2.4. Deep Learning Architectures for Multimodal Tea Evaluation

Deep learning characterizes complex nonlinear mappings between input features and quality indicators by constructing multilayer neural networks, and has become an important technical foundation for intelligent tea evaluation [16,17,18]. In tea-related applications, representative models include DNNs, CNNs, RNNs, attention mechanisms, graph neural networks, and other cross-modal deep architectures [35].

DNNs are more suitable for nonlinear classification and regression modeling based on structured feature matrices, such as the mapping between preprocessed and variable-selected spectral features and physicochemical indicators [36]. Compared with traditional linear regression or shallow machine-learning methods, DNNs can learn higher-order relationships among multiple constituents, nonlinear absorption responses, and quality labels. However, their performance is highly dependent on sample size, annotation quality, and regularization strategies. For most tea-related studies, in which sample sizes remain limited, DNNs are more appropriately used as moderately complex feature learners rather than as unconstrained networks that are simply deepened without limitation.

CNNs are particularly effective for processing images, hyperspectral data, and two-dimensional spectral maps, and have been widely applied to tea appearance grading, liquor color evaluation, defect recognition, and spatial-distribution visualization [25]. For one-dimensional spectral data, 1D-CNNs can also automatically learn local wavelength patterns, thereby reducing reliance on manually selected characteristic wavelengths. For HSI data, the core value of CNNs lies in their ability to simultaneously extract spatial and spectral information, enabling models not only to classify samples into quality grades but also to visualize the spatial distribution of chlorophyll, moisture, or other indicators at the pixel level [29].

RNNs and their variants are more suitable for processing time-series signals generated during dynamic tea processing. Tea fermentation, drying, scenting, and aging are not static state-recognition problems; rather, they are time-series processes involving continuous changes in color, aroma, moisture, and chemical composition. Therefore, RNNs, LSTMs, GRUs, or networks with temporal attention are suitable for predicting fermentation endpoints or processing stages [37,38]. If temperature and humidity, image data, VOC signals, and spectral signals are integrated into a unified temporal framework, it may also be possible to construct an approximate causal representation linking processing state to quality outcome, which is particularly important for online process control.

Attention mechanisms, graph neural networks, and lightweight modeling strategies should be regarded as necessary components of multimodal tea-sensing deployment rather than optional add-on modules. Attention mechanisms can be used to identify key wavelengths, critical imaging regions, important sensor channels, and decisive time points, thereby improving interpretability and suppressing irrelevant noise [20]. Graph neural networks are more suitable for representing structural relationships among modalities, indicators, and processing stages. For industrial applications, models must also balance lightweight deployment with interpretability. Therefore, compact convolutional networks, knowledge distillation, pruning, quantization, sparse attention, hybrid chemometric–deep learning frameworks, and interpretation strategies based on attention heatmaps or feature-contribution ranking should be incorporated as constraints during model design, rather than treated as remedial measures after deployment [19,20].

### 2.5. Emerging Sensor Models and Image-Feature Techniques

In addition to commonly used modalities such as machine vision, NIR/MIR, HSI, Raman spectroscopy, fluorescence, and electronic nose/electronic tongue systems, several emerging sensing platforms may further expand the representational capacity of future multimodal tea-sensing systems. First, prism-coupled surface plasmon resonance (prism-coupled SPR/PRISM) and photonic crystal fiber surface plasmon resonance sensing (PCF-SPR) show potential for refractive-index change detection and highly sensitive interfacial response measurement. Conventional prism-coupled SPR can be traced back to the classical optical excitation of surface plasmons based on frustrated total reflection [39], whereas PCF-SPR introduces surface plasmon resonance responses into microstructured optical fiber platforms, thereby further expanding the design space of SPR sensors in terms of compactness, coupling flexibility, and highly sensitive interfacial detection [40]. Although these platforms have not yet become mainstream tools for tea quality evaluation, they are worth being prospectively included in this review for the coupled detection of specific markers, adulterants, contaminants, or biochemical recognition elements.

IoT-based sensor models provide an engineering pathway for tea evaluation to move from “single offline measurement” toward “online continuous monitoring”. In tea processing, temperature, humidity, airflow, images, color, VOCs, and near-infrared signals essentially constitute a multisource spatiotemporal system rather than a set of independent one-time measurements. An IoT-based tea fermentation monitoring system integrating Raspberry Pi, cameras, and wireless transmission has already been applied in tea-factory scenarios for the recognition of fermentation stages in black-tea-type processing, and real-time discrimination performance was improved through CNNs and majority voting [41]. Such studies indicate that the value of IoT platforms lies not in replacing a single laboratory instrument, but in integrating dispersed visual, environmental, and quality-related signals into an online monitoring network that can be accessed in real time.

In addition, GLCM and its combination with LBP and color-space features should be regarded as interpretable visual features that remain valuable in the deep-learning era. Ramola et al. systematically summarized the applicable boundaries of statistical texture-analysis methods, including GLCM, LBP, and ACF, and indicated that GLCM remains one of the most robust and interpretable methods for surface texture analysis [23]. More importantly, Tang et al. demonstrated in tea classification that the LBP–GLCM combination can effectively extract texture features from green tea leaves at relatively low computational cost, making it suitable for automated production-line environments [24]. Therefore, for samples with pronounced differences in surface structure, such as strip-shaped tea, granular tea, and matcha powder, GLCM should not be regarded as an outdated feature replaced by CNNs, but rather as a lightweight visual-modality supplement that can be fused with deep features to improve interpretability.

### 2.6. Dataset Standardization, Model Generalization, and Industrial Deployment

Although multimodal sensing and deep learning have shown considerable potential for tea quality evaluation, their industrial translation is still constrained by multiple bottlenecks related to data, instruments, models, and application scenarios. The primary challenge is the lack of standardized, large-scale, and reusable multimodal tea datasets. Tea samples differ in cultivar, geographic origin, season, plucking standard, processing technology, storage history, and grade definition. In addition, the sensory scoring systems, physicochemical measurement protocols, instrument types, acquisition illumination, and sample presentation methods used across different studies are not standardized [19,20]. As a result, models reported as having “high accuracy” in different studies are often obtained under local experimental conditions rather than under truly transferable data distributions.

This inconsistency directly affects model evaluation and generalization. Small and weakly standardized datasets increase the risk that models learn acquisition conditions, batch differences, or background noise rather than robust quality features. Random split validation within the same batch, origin, or instrument can also overestimate real-world performance. Moreover, chemical indicators are usually continuous variables, whereas sensory grades are often subjective and discrete; without unified annotation standards, multimodal fusion may learn label noise rather than true modality complementarity.

Vision systems are strongly affected by illumination, background, camera angle, and sample stacking. NIR/HSI systems are influenced by light-source aging, detector drift, temperature variation, and sample presentation. Electronic noses are susceptible to humidity, memory effects, and MOS sensor drift, whereas electronic tongues face electrode fouling, matrix effects, and declining long-term repeatability [27,40]. Therefore, sensor drift correction, instrument standardization, and calibration transfer must be regarded as fundamental components of multimodal tea-sensing systems rather than post-processing options. For cross-instrument NIR/spectral models, classical multivariate instrument standardization, including subsequent PDS-related approaches, provides a methodological basis for establishing transferable calibration relationships between different instruments [41]. In the context of tea evaluation, this means that if the same quality model is to be transferred from a laboratory platform to portable devices or factory online instruments, standard samples, transfer sets, and periodic recalibration procedures must be explicitly designed.

For most tea factories or industrial laboratories, practical quality control usually relies on one or two routine methods, such as sensory evaluation, moisture or color measurement, or selected physicochemical assays, whereas GC–MS, LC–MS, and standard wet-chemistry methods mainly serve confirmatory and calibration functions [2,3,14]. A realistic industrial pathway is therefore not to replace all existing laboratory workflows, but to build a hierarchical system of rapid screening, process monitoring, and laboratory confirmation. Machine vision, portable NIR, electronic noses, and IoT sensors can support high-throughput or online monitoring, while more accurate laboratory methods can provide calibration, risk rechecking, and regulatory confirmation.

**Figure 2:**
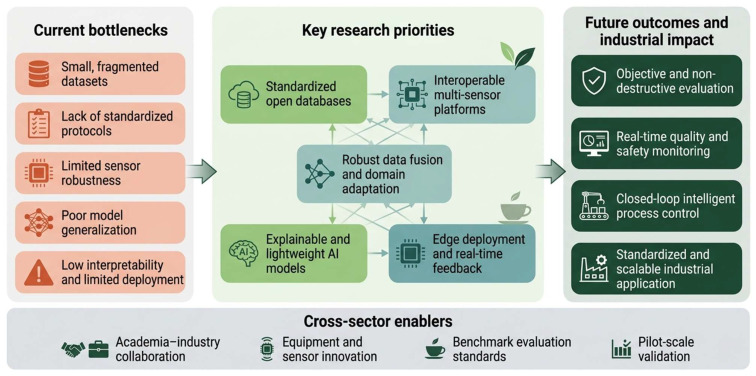
Future roadmap for multimodal intelligent tea quality and safety evaluation.

Deployable systems must balance accuracy, computational cost, and interpretability. High-accuracy deep models often require greater computing power and more complex parameter tuning than factory or portable-device environments can support. Lightweight models, edge deployment, model compression, and hybrid chemometric-deep learning frameworks should therefore be considered early. Industrial users also need to understand which wavelengths, image regions, gas-sensor channels, or electrochemical responses drive a prediction. Only interpretable, calibratable, and transferable systems are likely to move from proof-of-concept studies to routine quality control. In summary, a multimodal intelligent framework for tea quality evaluation should follow a unified logic: define quality attributes and target substance groups, select complementary sensing modalities, generate stable predictions through appropriate fusion strategies and deployable models, and support industrial translation through standardized datasets, sensor calibration, and hierarchical confirmation systems (Figure 2).

## 3. Applications in Representative Tea Products

### 3.1. Green Tea

Green tea is an unfermented tea valued for the balance between external appearance and intrinsic sensory quality, including emerald-green dry leaves, bright yellow-green liquor, fresh aroma, and brisk, umami-rich taste. Traditionally, these attributes have been assessed by experienced tea makers, which introduces unavoidable subjectivity. Multimodal sensing now provides a more objective basis for green-tea quality evaluation.

#### 3.1.1. Appearance Quality Monitoring

Integrated machine vision and spectral sensing have been explored for real-time monitoring of green-tea processing. Lan et al. combined a miniature NIR sensor with a visible-light camera to predict moisture content during the kill-green stage in real time [42]. After comparing partial least squares (PLS), support vector machines (SVMs), and neural-network models, the best performance was obtained with a whale-optimized Elman network using mid-level fusion features, yielding a validation correlation coefficient (Rp) of 0.9984. This study demonstrates that sensor fusion can support precise thermal control and improve consistency in final leaf appearance. In another example, Li et al. investigated ultrasound-assisted partial fermentation and used a colorimetric sensor array coupled with a CNN to track dynamic changes in volatile compounds and polyphenols [43]. Under optimized conditions, polyphenol degradation exceeded 66%, and the CNN outperformed conventional multivariate calibration. Chen et al. further showed that CNN models integrating fluorescence spectral features could non-destructively distinguish green-tea samples treated with different pesticides, with a test accuracy above 99% [28]. Collectively, these studies highlight the potential of multimodal sensing for both process monitoring and safety evaluation in green-tea production.

#### 3.1.2. Intrinsic Composition and Sensory Quality Prediction

Green-tea quality is also determined by infusion taste and chemical composition. Near-infrared (NIR) spectroscopy has therefore been widely used for rapid, non-destructive quantification of key constituents [44]. Wu et al., for example, developed a portable NIR system combined with partial least squares regression (PLSR) to predict caffeine and amino acid contents in green-tea infusion within minutes, with R^2^ values above 0.90 [35]. Electronic-tongue systems have likewise been used to evaluate taste attributes such as umami and astringency. When cyclic-voltammetry signals were reduced by principal component analysis (PCA) and input into a back-propagation neural network (BPNN), green teas of different quality grades could be classified accurately on the basis of taste [45]. Hyperspectral imaging has also been applied to green-tea grading. Early work showed that selecting three optimal wavelengths from visible-to-shortwave-infrared data, together with texture analysis, allowed an SVM model to classify green tea into five grades with accuracies of 98% and 95% in the training and test sets, respectively [46]. Despite these promising results, most multimodal studies on green tea remain at the laboratory stage, and models based on spectral or electronic-nose data are rarely validated across large sample sets or multiple geographic origins [47,48,49]. Future work should therefore focus on larger datasets, standardized evaluation frameworks, and more rigorous external validation.

### 3.2. Black Tea

Black tea undergoes full fermentation, and its quality depends strongly on the fermentation degree and volatile-aroma composition [50]. Conventional black-tea evaluation still relies heavily on subjective judgment of appearance, aroma, and taste. Multimodal sensing offers a route toward more objective and reproducible quality standards.

#### 3.2.1. Intelligent Aroma Quality Discrimination

Electronic-nose systems have been widely used to classify black-tea aroma types and grades because they can rapidly capture overall volatile fingerprints. Wang et al., for instance, combined a metal-oxide-semiconductor (MOS) e-nose with automated thermal desorption GC–MS to analyze volatile profiles in Keemun black tea of different grades, and achieved successful discrimination using partial least squares-discriminant analysis (PLS-DA) [2]. The e-nose was sensitive to changes in overall aroma intensity associated with fermentation and could distinguish low-, medium-, and high-grade samples. Its limitation, however, lies in the identification of trace compounds that define subtle aroma nuances. For this reason, e-nose screening is often complemented by chromatographic confirmation. Recent work has increasingly adopted a combined strategy of rapid e-nose screening plus chromatographic/olfactometric identification, enabling more comprehensive evaluation of premium black-tea aroma [2,3].

#### 3.2.2. Rapid Determination of Chemical Composition

Key chemical indices of black-tea quality, such as theaflavins and thearubigins, have traditionally been determined by laborious wet-chemistry methods [51,52,53,54]. Portable spectroscopic tools, especially NIR, now offer a faster and non-destructive alternative. Handheld NIR combined with spectral preprocessing and PLSR has been used to predict theaflavin content in tea infusion, achieving calibration R^2^ values of up to 0.94 and prediction errors below 5% [35]. Visible-light machine vision has also been used to evaluate liquor color and infused-leaf appearance by extracting Lab parameters from digital images and correlating them with sensory scores [55]. In addition, FT-NIR combined with chemometric algorithms has been used to assess both taste attributes and internal composition. A BP-AdaBoost model, for example, predicted eight taste-related compounds in black tea with prediction-set correlation coefficients above 0.76, outperforming the conventional Si-PLS model [56]. Surface-enhanced Raman spectroscopy (SERS) is another emerging tool for rapid assessment of intrinsic quality markers; AuNP-based SERS substrates coupled with a Si-GA-PLS model enabled rapid prediction of caffeine content in black tea [57]. Electronic-tongue systems have also shown value in taste evaluation. A cyclic-voltammetry electronic tongue integrated with a Si-VCPA-PLS model predicted total free amino acids with Rp = 0.84 after multi-electrode data fusion [58]. Together, these approaches illustrate the growing feasibility of continuous, non-destructive monitoring of black-tea chemistry.

#### 3.2.3. Fermentation Process Monitoring

Fermentation is a decisive step in black-tea manufacture and is traditionally controlled on the basis of experience with time, temperature, humidity, and aroma. Multimodal sensing provides a more objective basis for monitoring and regulating this process. Electronic noses can track dynamic aroma changes released during fermentation, and signal stabilization may indicate that fermentation is approaching completion [37,59]. Raman spectroscopy has likewise been used to monitor chemical transformations such as polyphenol oxidation and pigment formation by tracking changes in characteristic bands. Although intelligent monitoring of black-tea fermentation is still at an early stage, related studies in oolong and Pu-erh tea demonstrate the feasibility of this approach through aroma-pattern recognition and polyphenol prediction using sensor arrays and deep learning [38]. A recent study on black-tea pile fermentation further developed a portable artificial olfactory system based on a printed colorimetric sensor array and a KNN-AdaBoost model, achieving 100% classification accuracy in both training and prediction sets for fermentation-stage discrimination [60]. These results underscore the potential of real-time, non-invasive sensing for intelligent control of black-tea fermentation.

### 3.3. Dark Tea

Dark tea, a representative post-fermented tea category, includes products such as Pu-erh from Yunnan and Anhua dark tea from Hunan. Its distinctive sensory profile is shaped by pile fermentation (wo dui) and long-term aging, resulting in reddish-brown liquor, aged aroma, and a thick, mellow mouthfeel [61,62]. Traditional evaluation depends largely on expert olfactory and gustatory judgment of fermentation degree and aging status. Multimodal sensing provides a promising route toward more objective and quantitative assessment of these complex quality traits.

#### 3.3.1. Intelligent Identification of Aroma and Taste Qualities

During fermentation and aging, dark tea develops characteristic aged aromas and a mellow taste profile. Electronic-nose systems can capture VOC fingerprints that change across fermentation stages and storage years, enabling pattern recognition and sample classification. Electronic-tongue systems similarly mimic taste perception through liquid sensor arrays and can quantify infusion taste characteristics. For example, voltammetric e-tongue data combined with deep learning have been used to discriminate Pu-erh teas with different storage durations [38]. Ouyang et al. further integrated voltammetric e-tongue signals with a BP neural network to predict total free amino acids in dark-tea samples, achieving Rp = 0.84 [59]. These olfactory and gustatory sensing strategies provide a data-driven basis for standardizing aroma and taste evaluation in dark tea.

#### 3.3.2. Internal Components and Quality Indicator Detection

Dark-tea quality is also closely related to chemical changes during fermentation, including polyphenol oxidation and variations in caffeine and organic-acid content. Non-destructive methods such as NIR and Raman spectroscopy therefore offer valuable tools for rapid assessment of internal quality attributes. Related FT-NIR studies coupled with chemometric modeling have shown that multiple taste-related compounds can be predicted simultaneously with correlation coefficients above 0.85 in calibration and validation sets [63]. Optical colorimetric sensing offers another useful route. Liu et al. developed a colorimetric sensor array comprising eight porphyrins and one pH indicator to monitor chemical changes during Pu-erh pile fermentation; a CNN trained directly on the resulting color images achieved a prediction correlation close to Rp = 0.87 for total polyphenol content [38]. These spectral and colorimetric strategies provide efficient and objective tools for monitoring compositional change and supporting standardized quality evaluation in dark tea.

#### 3.3.3. Fermentation and Aging Process Monitoring

Pile fermentation is a key determinant of dark-tea quality. Traditionally, producers judge fermentation progress and endpoint from empirical cues such as heap temperature and aroma, which can introduce inconsistency. Multimodal sensing offers a basis for real-time monitoring and more precise process control. Sharmilan et al., for example, developed an artificial olfactory system for tea fermentation using an array of MOS gas sensors to capture odor characteristics at different stages; when combined with machine-learning algorithms, the system achieved nearly 100% accuracy in stage classification [64]. Although this study was not limited to dark tea, it illustrates the feasibility of continuously tracking odor evolution to infer fermentation degree and optimize process endpoints. As sensor stability improves and fermentation datasets accumulate, intelligent online monitoring of dark-tea fermentation and aging should become increasingly practical.

Overall, multimodal sensing can convert traditionally experience-based evaluation of dark-tea fermentation and post-fermentation aging into quantitative, data-driven monitoring. This transition is important for standardizing product quality and improving process control in large-scale production.

### 3.4. Matcha

Matcha is produced by steaming and drying tender tea leaves to obtain tencha, which is then milled into a fine powder. Its quality is determined not only by sensory traits such as bright green color, fresh aroma, and umami taste, but also by the content and spatial uniformity of key components, including chlorophyll and amino acids [65,66].

#### 3.4.1. Visualized Analysis of Color and Composition

Hyperspectral imaging (HSI) is particularly attractive for matcha because it simultaneously acquires spectral and spatial information, enabling visualization of the distribution of quality-related variables. Ouyang et al. used hyperspectral microscopic imaging (HMI) in the 400–998 nm range to predict color and physicochemical indices in matcha samples [23]. After feature selection by competitive adaptive reweighted sampling (CARS) and random forest, combined with PLSR modeling, they achieved accurate prediction of total chlorophyll content and chromaticity, with a best prediction correlation of Rp = 0.8093. Pixel-wise application of the optimized model generated full-field maps of chlorophyll and other indicators, revealing spatial heterogeneity in color and composition and thereby providing information relevant to grinding efficiency and product consistency. VNIR hyperspectral approaches have also been used for simultaneous quantification of caffeine, tea polyphenols, and free amino acids using standard normal variate (SNV) preprocessing, CARS/BOSS feature selection, and PLS modeling [67]. These studies demonstrate the promise of hyperspectral methods for objective matcha-quality analysis.

#### 3.4.2. Volatile Aroma and Grade Identification

Traditional assessment of matcha aroma lacks rapid and quantitative tools [68]. To address this gap, researchers have developed odor-sensing systems for grade discrimination. Ouyang et al. designed a ZIF-8-assisted nano colorimetric sensor array to capture aroma fingerprints of matcha and combined it with density functional theory (DFT) to interpret the sensing mechanism [69]. By incorporating pH indicators and metalloporphyrins within ZIF-8, the array achieved stronger selective adsorption of VOCs. Compared with the unmodified array, the ZIF-8-enhanced platform increased color-response intensity by 1.13–4.75 times and improved classification performance; among the tested algorithms, a BP-ANN achieved the best prediction accuracy (95%), representing a 7.5% improvement over the conventional array. DFT analysis supported the high affinity of the ZIF-8 porous structure for key aroma compounds. In another study, Zhang et al. developed a COF@MOF-based colorimetric sensor combined with AI-assisted analysis to visualize VOC changes during matcha drying, with 95.74% accuracy for the identification of seven drying stages under 20–90% relative humidity [70]. These results highlight the value of nanomaterial-enhanced sensing arrays and intelligent algorithms for rapid aroma evaluation of matcha.

#### 3.4.3. Quality Monitoring During Processing

The drying stage of tencha strongly influences the color and nutritional quality of the final matcha product. Whereas drying has traditionally been judged visually, multimodal sensing now enables real-time monitoring of key indicators. One study combined visible–near-infrared (Vis-NIR) spectroscopy with a colorimetric sensor array to classify matcha grades based on VOC-induced color responses [71]. This non-invasive strategy supports continuous monitoring of moisture and aroma-related compounds during processing. Portable NIR systems have also been used to monitor carotenoid content during drying; after variable selection and PLS modeling, one study achieved Rp = 0.9592, confirming the feasibility of tracking pigment precursors associated with color and aroma [35]. Likewise, Vis-NIR spectroscopy fused with colorimetric-sensor-array data has been used to evaluate aroma quality during tencha drying, achieving classification accuracies of 94.68% and 93.48% in the training and prediction sets, respectively, while also identifying key VOCs such as pentanal [72]. These studies demonstrate the potential of multimodal sensing for real-time regulation of moisture, pigments, and aroma during matcha processing.

From raw-material drying to finished-product grading and spatial visualization of powder quality, multimodal sensing has been applied across multiple stages of matcha quality control. These studies provide an increasingly objective basis for matcha evaluation and lay the groundwork for more intelligent production. In addition, physicochemical indicators in matcha show strong associations with sensory quality, suggesting that integrated models based on selected key variables can support effective sensory evaluation [26,73]. Continued advances in sensor design and machine-learning algorithms should further improve the precision and practicality of matcha-quality monitoring.

### 3.5. Jasmine Tea

Jasmine tea is a reprocessed scented tea produced by repeatedly scenting a green- or black-tea base with fresh jasmine flowers. Its quality is judged primarily by aroma intensity and aroma purity. Traditional evaluation therefore depends heavily on expert olfactory assessment. Multimodal sensing offers a promising route toward more objective evaluation of jasmine-tea aroma quality.

#### 3.5.1. Aroma Intensity and Purity Detection

Electronic noses have been widely used for rapid classification of jasmine-tea aroma profiles. Wang et al. combined e-nose measurements with automated thermal desorption GC–MS to compare volatile components among jasmine teas of different grades and achieved successful classification with PLS-DA [2]. The e-nose effectively distinguished differences in overall aroma intensity associated with the number of scenting rounds, such as once-, three-times-, and six-times-scented teas. However, its sensitivity to trace compounds responsible for the delicate aroma nuances of premium jasmine tea was limited [2]. This suggests that e-nose systems are well-suited to rapid screening of aroma strength, but less effective for fine discrimination of aroma type unless they are combined with more specific analytical tools such as GC–MS.

To overcome the limits of single-sensor systems, recent studies have explored multimodal strategies for jasmine-tea aroma evaluation. Gas chromatography-ion mobility spectrometry (GC–IMS), for example, can rapidly generate aroma fingerprint maps. In one study, jasmine teas subjected to one to six scenting rounds were clearly separated on the basis of their volatile fingerprints, especially after PCA [74]. GC–MS coupled with gas chromatography-olfactometry (GC–O) has further helped identify characteristic aroma compounds associated with different scenting methods. For example, single-petal jasmine-scented teas contained higher levels of α-farnesene, jasminelactone, and indole, which contributed to richer and sweeter notes, whereas double-petal jasmine teas contained more methyl benzoate, associated with a fresher floral aroma [75,76]. Such findings show that multimodal chemical profiling can provide a more detailed basis for quantitative aroma evaluation.

#### 3.5.2. Intelligent Control of the Scenting Process

The floral character of jasmine tea is established through repeated scenting cycles in which fresh jasmine flowers are layered with the tea base. Decisions on flower dosage and scenting duration are still largely guided by artisanal experience. Multimodal sensing offers new opportunities to monitor aroma adsorption dynamically and optimize this process. One proposed strategy is to use an electronic nose after each scenting round to capture aroma-intensity profiles and feed them into a regression model that estimates the deviation from an optimal aroma target, thereby guiding whether further scenting is required [1]. Supporting this idea, An et al. used GC–MS to compare jasmine teas produced with different numbers of scenting rounds and found that characteristic compounds such as phenylethanol, jasmone, and indole increased progressively before approaching a plateau after the fifth or sixth round [77]. These compounds could therefore serve as practical indicators for rapid process monitoring. With larger datasets and more robust prediction models, intelligent control of jasmine scenting should become feasible, helping avoid both insufficient aroma uptake and over-scenting.

#### 3.5.3. Evaluation of Appearance Quality

Besides aroma, the quality of jasmine tea also involves appearance traits such as uniformity and the presence of flower debris. Machine vision could be used to evaluate whether the dried product retains a desirable glossy green appearance and whether residual petals are excessive [12]. Although this area remains underexplored, methods developed for green and black tea image analysis can be adapted readily. Through image segmentation and extraction of color and morphological features, the visual quality of jasmine tea could be assessed more objectively against grading standards.

Table 2 summarizes the main sensing modalities, target indices, and modeling methods used for quality evaluation across green tea, black tea, dark tea, matcha, and jasmine tea. Overall, multimodal studies on jasmine tea remain limited but show clear potential. Combining rapid odor sensing with deep-learning classification may enable automated evaluation of aroma intensity and purity and support more standardized grading of scented teas.

### 3.6. Safety-Related Extensions: Rapid Screening of Pesticide Residues

Pesticides are widely used in tea cultivation, and excessive residues represent an important food-safety concern [78,79]. Conventional chromatographic and mass-spectrometric methods remain the reference approaches because of their sensitivity and reliability, but they are time-consuming and less suitable for high-throughput or on-site screening. Therefore, rapid sensing methods combined with chemometric or deep-learning algorithms have attracted increasing attention as complementary tools for preliminary tea-residue screening (Table 3).

Spectroscopic and spectral-imaging methods currently represent the main route for rapid pesticide-residue detection in tea. Fluorescence hyperspectral imaging combined with feature selection and 1D-CNN/random-forest modeling has been used to identify multiple pesticide residues on tea-leaf surfaces, achieving a test-set classification accuracy of 99.05% [28]. Handheld Raman or SERS platforms coupled with deep-learning models have also shown potential for on-site pesticide identification in tea matrices [80]. In addition, the fusion of NIR and SERS spectra can combine the broad compositional information provided by NIR with the high molecular specificity of SERS, thereby improving quantitative detection performance in complex samples [81].

Nanomaterial-assisted sensing further improves the sensitivity of residue detection. SERS substrates based on Au@Ag nanostructures, Au-Ag OHCs, or aptamer-assisted gold nanoparticles have been reported for the qualitative and quantitative detection of thiram, pymetrozine, imidacloprid, 2,4-D, chlorpyrifos, acetamiprid, and other pesticide residues in tea or matcha samples [82,83,84,85]. Upconversion-fluorescence and FRET-based platforms have also enabled sensitive detection of heavy-metal ions and organophosphorus pesticides such as diazinon and malathion [86,87,88]. However, pesticide detection involves highly diverse targets, complex matrix effects, and different regulatory requirements. In this review, residue detection is therefore treated as a safety-related extension rather than the central focus. Future work should emphasize standardized residue databases, miniaturized sensing devices, robust external validation, and integration with laboratory confirmatory methods.

## 4. Actionable Methodological Roadmap for Multimodal Tea Quality Evaluation

Although multimodal sensing and deep learning provide a promising framework for tea quality evaluation, future studies should move beyond proof-of-concept model construction and adopt more standardized, reproducible, and deployment-oriented methodological pipelines. A practical roadmap should address not only sensor selection and model construction, but also dataset curation, leakage-free validation, uncertainty assessment, model interpretation, and independent external testing.

### 4.1. Dataset Curation, Harmonization, and Leakage-Free Validation

The first requirement is to establish standardized and reusable multimodal tea datasets. Each tea sample should be accompanied by complete metadata, including tea type, cultivar, geographic origin, harvest year, season, plucking standard, processing batch, storage condition, and grade definition. Sensor-related metadata should also be recorded, including instrument type, spectral range, spatial or spectral resolution, illumination condition, sample presentation mode, calibration status, and acquisition protocol. For multimodal studies, all data modalities, such as RGB images, spectral curves, hyperspectral cubes, electronic-nose responses, electronic-tongue signals, and reference physicochemical measurements, should be linked through a unified sample identifier. Dataset splitting should be performed at the sample, batch, origin, or harvest-year level rather than at the pixel, spectral-replicate, or repeated-measurement level, because inappropriate splitting can lead to data leakage and overestimated model performance.

For internal validation, nested cross-validation should be recommended when preprocessing optimization, feature selection, or hyperparameter tuning is involved. In this design, the outer loop is used to estimate model generalization, whereas the inner loop is used for variable selection, model selection, and hyperparameter optimization. This strategy can reduce the bias caused by using the same data for both model selection and performance estimation [89,90]. Bootstrapping can further be used to quantify uncertainty and generate confidence intervals for performance indicators such as accuracy, R^2^, RMSE, AUC, sensitivity, specificity, or classification error [91]. In addition, Y-randomization should be performed as a negative-control test by randomly permuting the response labels and rebuilding the model using the same modeling workflow. If the randomized models still show high performance, the original model may reflect chance correlation, data leakage, or overfitting rather than a meaningful relationship between sensor signals and tea quality attributes [92]. Therefore, future multimodal tea studies should report not only single-split performance values, but also repeated validation results, uncertainty intervals, and negative-control tests.

### 4.2. Model Interpretability, External Validation, and Industrial Readiness

Model interpretability should be incorporated into multimodal tea-quality modeling from the beginning rather than treated as a post hoc supplement. For spectral models, global feature-importance metrics, permutation importance, SHAP values, VIP scores, or attention weights can be used to identify key wavelengths associated with tea polyphenols, caffeine, amino acids, pigments, moisture, or other quality-related constituents [93,94]. For machine-vision and hyperspectral-imaging models, saliency maps, Grad-CAM, attention maps, or pixel-wise contribution maps can help determine whether predictions are driven by meaningful regions, such as leaf surface, liquor color, powder distribution, or local defects, rather than by background or illumination artifacts [95]. For electronic-nose and electronic-tongue systems, sensor-channel importance analysis can reveal which gas sensors or electrochemical electrodes contribute most to aroma or taste discrimination. Local explanation methods such as LIME may also be useful for interpreting individual predictions and identifying abnormal samples or modality-specific failures [96]. In multimodal models, both modality-level and feature-level contributions should be evaluated to determine whether the model truly benefits from complementary information or is dominated by a single high-dimensional modality.

External validation should be based on truly independent samples that are excluded from all modeling steps, including preprocessing, normalization, feature selection, model training, and hyperparameter tuning. Preprocessing parameters, selected wavelengths, feature subsets, scaling coefficients, and model parameters should be fitted only on the training set and then applied unchanged to the independent test set. To realistically assess generalizability, external validation should be stratified by tea type, cultivar, geographic origin, harvest year, processing batch, storage condition, and instrument platform whenever possible. Recommended validation schemes include leave-one-origin-out, leave-one-year-out, leave-one-batch-out, and leave-one-instrument-out testing. Before industrial deployment, multimodal systems should also be evaluated under realistic operating conditions, including variations in illumination, sample stacking, humidity, sensor drift, instrument aging, operator handling, and online processing speed. Only models that remain interpretable, calibrated, transferable, and robust under these independent validation conditions can be considered ready for routine industrial tea-quality control.

## 5. Conclusions and Perspectives

Multimodal sensing combined with deep learning is providing an increasingly powerful framework for non-destructive tea quality evaluation. Compared with conventional sensory assessment and destructive laboratory analysis, these approaches enable more objective, rapid, and information-rich characterization of tea by integrating complementary signals related to appearance, chemical composition, aroma, taste, processing status, and safety-related screening. The main contribution of this review is to clarify the role of sensor complementarity, fusion strategies, and deployable model design in representative tea products. At the same time, this field remains at a relatively early stage of translation from laboratory research to practical application. Major constraints include small and weakly standardized datasets, limited external validation across tea categories and production scenarios, insufficient sensor stability under real operating conditions, underdeveloped multimodal fusion pipelines, and persistent challenges in model interpretability, transferability, and lightweight deployment. In particular, future studies should adopt leakage-free nested validation, uncertainty estimation, Y-randomization, interpretable feature-attribution methods, and independent validation schemes stratified by variety, origin, harvest year, and processing batch.

## Figures and Tables

**Table 2:** Comparison of multimodal quality evaluation of representative tea types in terms of key indicators, sensing modalities, and modeling methods.

Tea Type	Main Quality Indicators	Sensing Modalities	Modeling Methods	References
Green Tea	Appearance uniformity and color; moisture content; polyphenols; amino acids	Machine vision; near-infrared spectroscopy; colorimetric sensor arrays	PLS regression; Elman neural network; CNN-based prediction	[37,38]
Black Tea	Aroma-compound abundance; theaflavin content; fermentation degree	Electronic nose; visible/near-infrared spectroscopy; machine vision (liquor color)	MOS-based e-nose + PLS-DA; NIR quantification of theaflavins; image-based color evaluation	[2]
Dark Tea	Aged-aroma purity and richness; taste fullness; polyphenols; caffeine; fermentation degree; post-fermentation age	Electronic nose/electronic tongue; near-infrared/Raman spectroscopy; colorimetric sensor arrays	Odor-pattern recognition; NIR + PLSR compound prediction; colorimetric array + CNN for fermentation evaluation	[38,62]
Matcha	Powder color and greenness; free amino acids; aroma quality	Hyperspectral imaging; nano-enabled colorimetric arrays; spectroscopy-based fusion sensing	HMI + PLSR for chlorophyll prediction; ZIF-8 colorimetric array + ANN for grade classification	[69]
Jasmine Tea	Aroma intensity and purity; appearance uniformity; flower-debris content	Electronic nose; GC–IMS fingerprinting; machine vision	E-nose + PLS-DA for grade discrimination; GC–IMS fingerprint analysis; image-based impurity detection	[2]

PLS, partial least squares; Elman, Elman neural network; CNN, convolutional neural network; MOS, metal oxide semiconductor; PLS-DA, partial least squares-discriminant analysis; NIR, near-infrared spectroscopy; PLSR, partial least squares regression; HMI, hyperspectral microscopic imaging; ZIF-8, zeolitic imidazolate framework-8; ANN, artificial neural network; GC–IMS, gas chromatography–ion mobility spectrometry.

**Table 3:** Representative multimodal and advanced sensing methods for non-destructive detection of pesticide residues in tea.

Method	Sensing Modality	Model Performance	References
Fluorescence hyperspectral imaging + CNN/RF	EEM fluorescence + 1D-CNN	Classification accuracy: 99.05% for identification of multiple pesticide residues	[28]
Handheld Raman spectroscopy + deep CNN	SERS Raman + 1D-CNN	Multi-pesticide classification accuracy > 95%	[80,81]
NIR + SERS fusion	NIR reflectance + SERS	Pesticide quantification with fused PLSR model, R^2^ ≈ 0.99	[82]
Machine vision + electronic nose (conceptual)	Visible imaging + MOS gas sensors	Joint screening of suspicious residue spots and odor anomalies	[81]

EEM, excitation–emission matrix; 1D-CNN, one-dimensional convolutional neural network; CNN/RF, convolutional neural network with random forest; SERS, surface-enhanced Raman spectroscopy; NIR, near-infrared reflectance; PLSR, partial least squares regression; MOS, metal oxide semiconductor.

## Data Availability

No new data were created or analyzed in this study.
